# Mr. Franks, on the Brunonian System

**Published:** 1800-01

**Authors:** J. Franks

**Affiliations:** Smith street, Westminster


### *Mr. Franks, on the Brunonian Syſtem.*

[ Concluded from our laſt Number, pp. 449—452. ]

DR. FOTHERGILL obſerves, "It does not ſeem abſurd to compare the animal machine to a clock; let the wheels whereof be in ever ſo good order, the mechaniſm complete in every part, and wound up to the full pitch, yet without ſome impulſe communicated to the pendulum, the whole continues motionleſs."

With reſpect to the animal machine, the oxygen of the air may be conſidered as the impulſe, and the left auricle the living pendulum to which this impulſe is communicated.

From what has been obſerved on the ſtate of apparent death from a temporary ſuſpenſion of reſpiration, I am inclined to infer, that the exiſtence of excitability directly depends on the conſtant reſtoration of that degree of ſtimulant force derived from the air by the blood in the lungs, to which the left auricle and ventricle have been accuſtomed, and without which there there is a ſuſpenſion of excitement, and not on the powers of aſſimilation in the ſtomach, &c. The powers of aſſimilation, as well as the excitability, are entirely dependant on the ſtimulus of arterial blood.

---

OF PREDISPOSITION TO DISEASES OF THE SYSTEM.

Dr. WILSON objects to the idea of Prediſpoſition to Diſeaſes of the Syſtem,—page 519.—" The ſyſtem in general is either in a ſtate of health or diſeaſe. Prediſpoſition to general diſeaſe is a ſtate that cannot be defined. There is, perhaps, no part of the writings of Dr. BROWN more exceptionable than his Obſervations on Prediſpoſition to Diſeaſe. Theſe, however, as they do not properly form any part of his ſyſtem, it did not appear neceſſary to conſider here."

I am of opinion that prediſpoſition to general diſeaſe forms a part of the Brunonian doctrine, which deſerves conſideration; for it appears to me to be contrary both to reaſoning and matter of fact to conclude, that the ſyſtem is always either in a ſtate of health or diſeaſe. If from obſervation it is evident, that the ſyſtem in a ſtate of health this day cannot be precipitated into dropſy on the next, or on the third, fourth, or fifth day, by a ſubtraction from the degree of ſtimulus which had previouſly ſupported the body in a ſtate of health, but that by perſevering in the application of the debilitating powers for ſeveral months together, and we then find this diſeaſe make its appearance,—may we not conclude, that the ſyſtem has gradually receded from the ſtandard of health during the interval, and that prediſpoſition to ſome general diſeaſe of debility immediately commenced, (no matter in what form the deviation from health might be) when the daily allowance of animal food, wine, and porter were prohibited, and a vegetable diet with water for beverage ſubſtituted. If from obſervation we find, that there is an intermediate ſtate between health and diſeaſe, may not the word, Prediſpoſition, be employed to expreſs ſuch a ſtate of the ſyſtem? and ought we to object to it merely becauſe it cannot be defined?

It is not a ſtate eſſentially different from health or diſeaſe, only different ſo far as the excitement is concerned. It has an equal affinity with both, and may by management be readily converted into either.

But there appears ſomething like a contradiction in another part of this work with reſpect to Prediſpoſition, although it is poſſible that I do not rightly conceive the author's meaning; for, on referring to page 468, Dr. Wilſon obſerves, " Between the healthy ſtate in which the excitability and ſtimuli applied are are in due proportion, and death, in which the excitability is extinguiſhed either by an exceſs, or too great an abſtraction of ſtimuli, the Brunonian ſyſtem ſuppoſes all poſſible gradations. Theſe are evidently to be divided into two claſſes; thoſe in which the excitability is to a certain degree exhauſted by too great an application of ſtimuli, and thoſe in which a morbid accumulation is ſuppoſed to take place in conſequence of too great an abſtraction of ſtimuli. In the latter of theſe, the body is ſaid to be in a ſtate of direct, and in the former, of indirect debility. Theſe are ſuppoſed its morbid ſtates. *It is evident, however, that there is a ſtate of body different from either of theſe, and different alſo from the healthy ſtate.* When ſtimuli are too much abſtracted, debility is ſuppoſed immediately to enſue; but debility is not the immediate conſequence of too great an application of ſtimuli; the immediate conſequence of this is increaſed excitement."

The body in a ſtate of increaſed excitement, I apprehend to be the ſtate which Dr. Wilſon alludes to, as being different from a ſtate of debility, whether directly or indirectly induced, and different alſo from the healthy ſtate. But is not increaſed excitement, where ſthenic diatheſis always prevails, a ſtate of prediſpoſition to diſeaſe? If the excitement, and conſequently the diatheſis continue to be increaſed, a diſeaſe of the ſthenic claſs in one form or another will inevitably ſupervene; but if diminiſhed before the arrival of diſeaſe, a return of health may be the conſequence.

---

OF DIRECT AND INDIRECT DEBILITY.

Page 477.—Dr. WILSON aſks, " Are not the ſuppoſed ſtates of direct and indirect debility oppoſite conditions of body? Can we ſuppoſe them to produce preciſely the ſame train of ſymptoms? Yet Dr. Brown is conſtantly forced into this inconſiſtency."

I do not think that there exiſts any inconſiſtency in this inſtance. Direct and indirect debility ſeem to me to be a condition of body nearly one and the ſame, although a condition effected by oppoſite means. For, as health, prediſpoſition, and general diſeaſe, is the ſame condition of body, different only with reſpect to the excitement; ſo is direct and indirect debility the ſame ſtate or condition, different only with reſpect to the excitability; which in the former ſpecies is too much accumulated, and in the latter too much exhauſted.

Dr. BROWN having found from obſervation, that debility of the ſyſtem might be effected by oppoſite means, judged it proper to make a diviſion, and to employ the terms Direct and Indi- rect, rect, to give an idea of the manner in which debility had been induced in each particular caſe; that when the diſeaſes, of which it is the parent, became an object of our attention, the medical practice might be conducted on philoſophical principles. But it does not occur to me that Dr. BROWN ever intended to convey the idea, that direct and indirect debility are oppoſite conditions of the body, otherwiſe than with reſpect to the excitability.

But were we to admit, that which I am not inclined to admit, that direct and indirect debility are oppoſite conditions of body, from a knowledge of the equivocal nature of ſymptoms, ſuprize would not be excited, if we ſhould obſerve ſome of the ſymptoms to be the ſame in each of the conditions; for many of the ſymptoms of diſeaſe might be enumerated which occur equally in the ſthenic and aſthenic claſſes, as, delirium, pain, cough, thirſt, &c.

Dr. WILSON has noticed what he conceives to be another inconſiſtency in the Brunonian doctrine, with reſpect to debility; this is, the ſuppoſition that both ſpecies of debility may exiſt in the ſame body at the ſame time.

There is no difficulty in conceiving, that to a degree of exiſting debility directly induced, an additional degree may be ſuperadded indirectly, and vice verſa.

A perſon in whom the ſthenic is prevalent, after an exceſs in wine, experiences ſometimes on the following day, ſymptoms of debility, as, a pain in the head, nauſea, &c. It may be that he is his own phyſician on the occaſion, and the means which he employs to remove theſe ſymptoms, induce an additional degree of debility, eſſentially the ſame as that which had been previouſly induced by the abuſe of certain diffuſible ſtimuli; for it is a very common occurrence, that perſons ſo indiſpoſed preſcribe for themſelves emetics, cathartics, bleeding, and water-gruel. By an error of this ſort, a debility, which would have been only temporary, but for the application of directly debilitating powers, is often ſucceeded by a ſerious indiſpoſition.

I think that I have ſeen two inſtances, where the parties, who were both men of ſtrong conſtitutions, began to ſtimulate in exceſs very early in life, and continued in the practice for ſeveral years with impunity, with the exception of experiencing now and then ſome complaints from exceſſive excitement, but which were eaſily removed. At length, however, debility was very apparently induced; they were ſtill very ſtrong men when compared with others, but inferior in vigour to what they themſelves had been for ſeveral years before.

The already exiſting debility was at this time daily increaſed by by the abftraction of neceffary ftimuli, which in a very few months induced fuch a degree of debility as conftitutes death.

If, when the fyftem is in a ftate of debility indirectly induced, and in which the excitability is more or lefs exhaufted, the fum of ftimuli, which, by a continuance of application, induced, firft, exceffive excitement, and ultimately, the permanent debility, produces now a lefs degree of excitement than formerly, what fort of excitement will a confiderable reduction from this fum produce?

We may infer, and obfervation fanctions the inference, that in a very fhort time no excitement will arife, and death will take place, not from any thing pofitive, nor from the complete exhauftion of excitability, and often not through the intervention of any particular difeafe, but from the permanent fufpenfion of excitement, in confequence of a negation of the powers which fupport life.

If it fhould be required, what is the ftate of the excitability when one fpecies of debility is fuperadded to the other which is already eftablifhed,—Dr. Brown having afferted, that in one fpecies the excitability is too much accumulated, and in the other too much exhaufted,—I would anfwer, that the excitability is not otherwife permanently affected, than by receiving an addition to the morbid ftate of it, whether of accumulation or of exhauftion.

J. FKANKS.

